# Resveratrol Inhibits Enterovirus 71 Replication and Pro-Inflammatory Cytokine Secretion in Rhabdosarcoma Cells through Blocking IKKs/NF-κB Signaling Pathway

**DOI:** 10.1371/journal.pone.0116879

**Published:** 2015-02-18

**Authors:** Li Zhang, Yuanyuan Li, Zhiwen Gu, Yuyue Wang, Mei Shi, Yun Ji, Jing Sun, Xiaopeng Xu, Lirong Zhang, Jingtin Jiang, Weifeng Shi

**Affiliations:** 1 Clinical Laboratory, The Third Affiliated Hospital of Soochow University, Changzhou, Jiangsu 213003, P. R. China; 2 Oncology Laboratory, The Third Affiliated Hospital of Soochow University, Changzhou, Jiangsu 213003, P. R. China; UMR INSERM U866, FRANCE

## Abstract

Polydatin and resveratrol, as major active components in *Polygonum cuspidatum*, have anti-inflammatory, antioxidant and antitumor functions. However, the effect and mechanism of polydatin and resveratrol on enterovirus 71 (EV71) have not been reported. In this study, resveratrol revealed strong antiviral activity on EV71, while polydatin had weak effect. Neither polydatin nor resveratrol exhibited influence on viral attachment. Resveratrol could effectively inhibit the synthesis of EV71/VP1 and the phosphorylation of IKKα, IKKβ, IKKγ, IKBα, NF-κB p50 and NF-κB p65, respectively. Meanwhile, the remarkably increased secretion of IL-6 and TNF-α in EV71-infected rhabdosarcoma (RD) cells could be blocked by resveratrol. These results demonstrated that resveratrol inhibited EV71 replication and cytokine secretion in EV71-infected RD cells through blocking IKKs/NF-κB signaling pathway. Thus, resveratrol may have potent antiviral effect on EV71 infection.

## Introduction

Hand, foot, and mouth disease (HFMD), as a commensal disease, often occurs in children under the age of 10, and can be observed in adolescents and adults on occasion. The common pathogens of HFMD are EV71 and coxsackievirus A16 (CA16). EV71 belongs to the *Picornaviridae* family, and is characterized by a single-stranded positive RNA genome. Compared with CA16, EV71 often causes aseptic meningitis, encephalomyelitis, and acute flaccid paralysis, and even death [1, 2]. Generally, viruses can only survive in host cells, so it is difficult to develop effective drugs specifically against viruses without the damage on host cells. Currently, several kinds of drugs including interferons, antiviral chemicals, and vaccines have been developed to treat EV71 infection. However, the treatment efficacy of these developed drugs for EV71 infection is still not confirmed yet [3].

*Polygonum cuspidatum* (*P*. *cuspidatum*), a traditional Chinese herb, has been used as analgesic, anti-pyretic, diuretic and expectorant agents in clinical practice for long time. Resveratrol and polydatin (piceid), the major active components in *P*. *cuspidatum*, are confirmed to have pharmacological actions in cardiovascular protection, neuroprotection, anti-inflammation, immunoregulation, anti-oxidation, anti-tumor, and liver and lung protection [4]. Moreover, resveratrol reveals inhibitory activity on a wide variety of viruses including HIV-1 (human immunodeficiency virus), HSV-1 (herpes simplex virus), HSV-2, VZV (varicella-zoster virus) and HCMV (human cytomegalovirus), suggesting that this chemical agent can interfere with infection by altering signal pathways in cells rather than acting directly against the virus itself [5–8]. Polydatin is the most abundant form of resveratrol in nature, and can be extracted from grapes, peanuts, hop cones, red wine and chocolate products as well as many daily diets [9]. Previous studies have demonstrated that polydatin has many physiological properties such as anti-platelet aggregation, antioxidant activity of low-density lipoprotein (LDL), and cardioprotective, anti-inflammatory and immune-regulating functions [10–14]. However, the antiviral activity of polydatin on EV71 is unknown.

In order to clarify whether polydatin and resveratrol can inhibit EV71 replication, we have examined the cytotoxicity of resveratrol and polydatin on RD cells and evaluated their treatment efficacy on EV71 infection. Meanwhile, the total or phosphorylation levels of IKKs/NF-κB signaling pathway molecules have also been explored in the presence of resveratrol. The results demonstrated that resveratrol could effectively protect from EV71-infected RD cells and inhibit EV71 replication in a dose-dependent manner *in vitro*. In addition, resveratrol reduced the secretion of IL-6 and TNF-α by blocking the phosphorylation of IKKs/NF-κB signaling pathway molecules. However, polydatin was found to have weak inhibitory activity on EV71.

## Materials and Methods

### Chemicals, cells and antibodies

Polydatin (C_20_H_22_O_8_; MW, 390.39; purity, > 95%); resveratrol (C_14_H_12_O_3_; MW, 228.24; purity, 99%) were purchased from Aladdin Co., Ltd. (Shanghai, China). Ribavirin was provided by the National Institute for the Control of Pharmaceutical and Biological Products (Beijing, China). RD cells were purchased from Cell Bank of Type Culture Collection of the Chinese Academy of Sciences (Shanghai, China). DMSO was ordered from Borunlaite Co., Ltd. (Beijing, China). Dulbecco's modified Eagle's medium (DMEM) and fetal bovine serum (FBS) were purchased from Thermo Scientific HyClone (UT, USA). Hybond C membrane and ECL Western blot detection system were purchased from Pierce Company (Rockford, IL, USA). Rabbit polyclonal antibodies against IKKα, p-IKKα, IKKβ, p-IKKβ, IKKγ, p-IKKγ, IKBα, p-IKBα, NF-κB p50, p-NF-κB p50, NF-κB p65, p-NF-κB p65, and horseradish peroxidase (HRP) conjugated goat anti-rabbit IgG secondary antibodies were purchased from SAB Company (Pearland, TX, USA). Antibodies against anti-glyceraldehyde-3-phosphate dehydrogenase (GAPDH) were obtained from ProteinTECH Group (Chicago, IL, USA). Rabbit polyclonal antibody against EV71/VP1 was purchased from Abcam Company (Cambridge, UK).

### Virus isolation and propagation

EV71 strain GDV083 (ATCC VR-784) was provided by the China Center for Type Culture Collection (CCTCC) at the multiplicity of infection (MOI) of 2 in 4 ml of viral inoculum diluted with maintenance medium. RD cells were cultured in high glucose DMEM supplemented with 10% FBS at 37°C and in a humidified incubator with 5% CO_2_. When cells reached the confluence of 90%, the original media were removed and the monolayer cells were washed with PBS once. Approximately 1 × 10^6^ RD cells were incubated with EV71 at a MOI of 2 or as indicated and allowed for absorption for 1.5 h at 37°C. Unbound viruses were removed by washing the cells with medium, and 15 ml of maintenance medium was supplied again. Infected cells and culture supernatants were collected at different time intervals. When viruses were propagated up to the confluence of 90% in cell monolayer in MEM containing 2% FBS and antibiotics as described above, the viral titer was determined by cytopathic effect (CPE) and expressed as 50% of tissue culture infective dose (TCID_50_) per ml [15].

### Cytotoxicity assay of polydatin and resveratrol

The cytotoxicity of polydatin and resveratrol on RD cells was determined by quantifying the cell viability using MTT (3-[4.5-dimethylthiazol-2-yl]-2,5-diphenyl tetrazolium bromide; Sigma, USA) assay. In the assay, RD cells (3 × 10^5^ cells/well) were seeded on 96-well plates and incubated overnight at a 37°C incubator supplemented with 5% CO_2_. The medium was removed, and polydatin or resveratrol at various concentrations was added in triplicate. The diluted medium without the sample was used as the control. After 2 days of incubation at 37°C in a humidified incubator with 5% CO_2_, 10 μl of MTT solution [5 mg/ml in phosphate buffered saline (PBS)] was added to each well. After incubation at 37°C for 2 h, the supernatant was removed and 100 μl of DMSO was added to each well. After vigorous shaking, the absorbance was measured using a microplate reader (Bio-Rad, USA) at 570 nm. The 50% half cell toxicity (CC_50_) was calculated as the extract concentration necessary to reduce cell viability by 50% [16].

### The assay of antiviral activity

Totally 30 μl of suspension containing EV71 at a MOI of 2 and the resveratrol solutions with the serial two-fold dilution were added on the monolayer of RD cells in 96-well plates, and then incubated at 37°C for 1.5 h to allow attachment. Thereafter, the medium was aspirated from the well to remove the unabsorbed viruses. The cell monolayer was then washed with PBS and medium only with test samples at various dilutions were added to the plates, respectively. The concentrations of the test samples were equal or less than the maximal non-cytotoxic concentration. The virus-infected cells without drug treatment and the un-infected cells were used as the control. The study was performed in triplicate and all plates were incubated at 37°C in a humidified incubator with 5% CO_2_ and examined daily under an inverted microscope. The virus-induced CPE of the test samples was observed under a light microscope and compared with the virus and cell controls. When the untreated virus-infected cells showed 4^+^ CPE, the MTT assay as described above was performed. The inhibitory concentration for 50% (IC_50_) of dead cells relative to that of the virus control was estimated from the plots of the data.

### The time course analysis of polydatin and resveratrol on EV71 replication

Briefly, RD cells (3 × 10^5^ cells/well) were seeded and incubated for 24 h, and then infected with EV71 (MOI = 2) in the presence or absence of polydatin (200 μg/ml), resveratrol (30 μg/ml), and ribavirin (125 μg/ml), respectively. Polydatin, resveratrol and ribavirin were added at EV71 absorption for 1.5 h. Cell supernatants were collected at 0, 2, 4, 8, 12, and 24 h post-infection (p.i.), and EV71 titers were determined by TCID_50_.

### Cell extract preparation and Western blot analysis

EV71-infected cells were harvested as the above description. The pellets were washed and lysed with a lysis buffer (2% sodium dodecyl sulfate, 35 mM β-mercaptoethanol, 50 mM Tris-HCl [pH 6.8], and 1 mM phenylmethylsulfonylfluoride). Cell lysates were obtained by centrifugation at 13,000 rpm and 4°C. Total protein concentration was determined by the bicinchoninic acid protein assay kit (Pierce, USA). The proteins were then resolved by sodium dodecyl sulfate polyacrylamide gel electrophoresis (SDS-PAGE), and transferred to PVDF membrane (Millipore, USA). The membrane was blocked for 2 h with 5% nonfat dry milk solution in Tris-buffered saline containing 0.1% Tween-20, which was blotted with specific primary antibody, and followed by incubation with secondary antibody conjugated with horseradish peroxidase (Proteintech, USA). The immunoreactive bands were detected by ECL reagents (Pierce, USA) and developed by Super RX film (Fujifilm, Japan). The band intensity was quantified by ImageQuant densitometric analysis (Molecular Dynamics,USA).

### Detection of inflammatory cytokines

RD cells were infected with EV71 at a MOI of 2 at 37°C. Supernatants collected at different time points were clarified by centrifugation at 13,000 rpm. The concentrations of IL-2, IL-6 and TNF-α in culture supernatants from control, resveratrol, EV71 infection, and EV71 infection-pretreated with resveratrol groups were analysed by using luminex fluorescent technique through Milliplex magnetic beads (Millipore, Billerica, MA, USA) according to the manufacturer’s protocol. The level of fluorescence in each standard, quality control, and sample were detected with the FLEXMAP3D (Luminex Co., TX, USA). Data was subsequently analyzed using the MILLIPLEX Analyst V5.1 (VigeneTech Inc., Carlisle, MA, USA). Cytokine concentrations were obtained by interpolation of the standard curve using protein standards with stepwise five-fold dilution. Standard curve was established for each analyte with the Bio-plex manager software and the sample concentration was calculated from the standard curve.

### Statistical analysis

All data were presented as mean ± SD. Statistical analysis was conducted by SPSS 17.0 software and evaluated by ANOVA or Student’s t-test. A significant difference was considered as *p* value of less than 0.05.

## Results

### Cytotoxicity of polydatin and resveratrol

**Figure 1:**
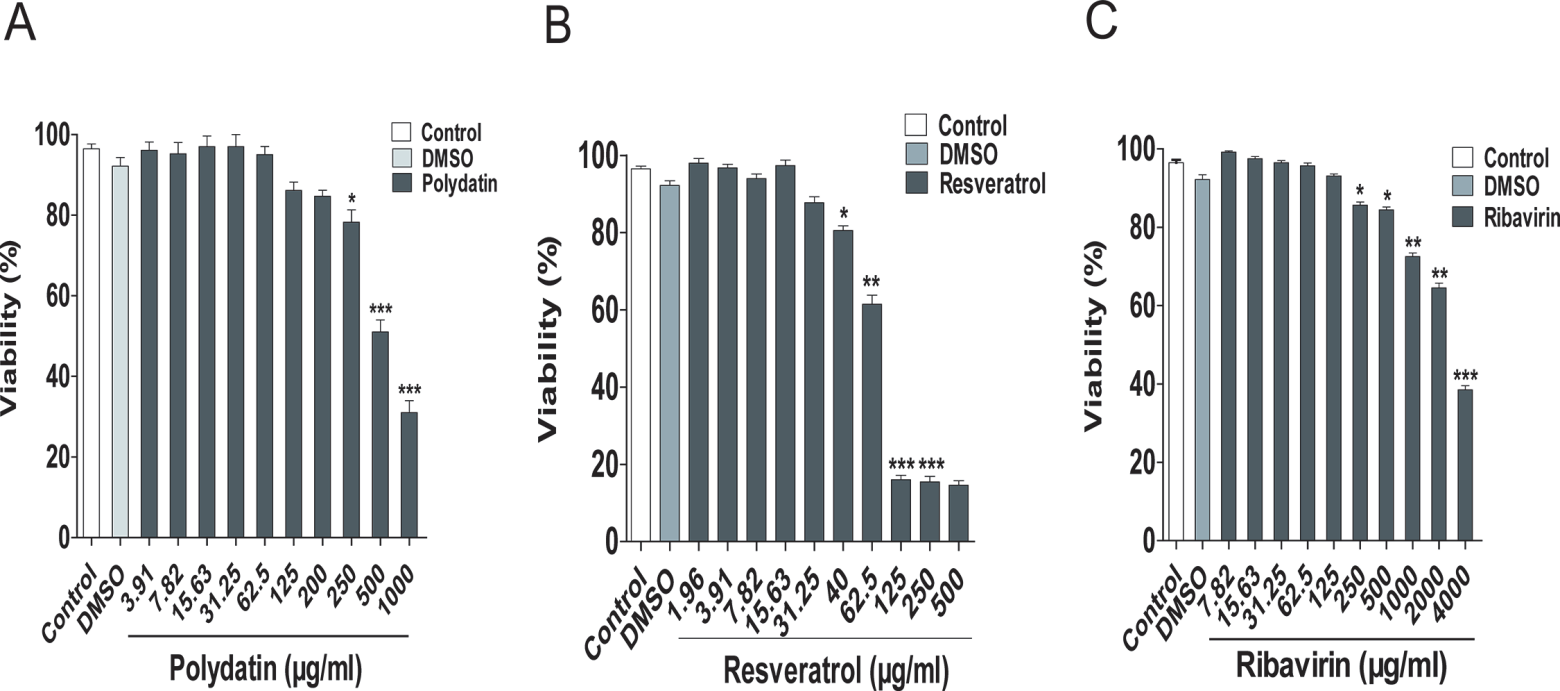
The cytotoxic effects of polydatin and resveratrol on RD cells. The cytotoxicity of polydatin **(A)**, resveratrol **(B)** and ribavirin **(C)** on RD cells were determined by quantifying the cell viability through MTT assay. The absorbance was measured using a microplate reader at 570 nm. The CC_50_ value was calculated as the extract concentration necessary to reduce cell viability by 50%. Data were expressed as mean ± SE from three independent experiments and analyzed by one-way ANOVA (*p < 0.05, **p < 0.01, ***p < 0.001).

To understand whether the inhibitory activity of polydatin and resveratrol on EV71 replication is associated with cytotoxicity, the viability of RD cells subjected to the treatment of polydatin, resveratrol or ribavirin for 2 days was determined. Mock treatment with DMSO did not result in the change in cell viability. Compared with the control cells, polydatin, resveratrol or ribavirin at the concentrations up to 200, 31.25 and 125 μg/ml did not reveal any cytotoxicity on RD cells (**Fig. 1A, 1B and 1C**). The CC_50_ of polydatin, resveratrol and ribavirin were 542.76, 85.73 and 3577.03 μg/ml, respectively.

### The antiviral efficiency of polydatin and resveratrol on EV71 replication

**Figure 2:**
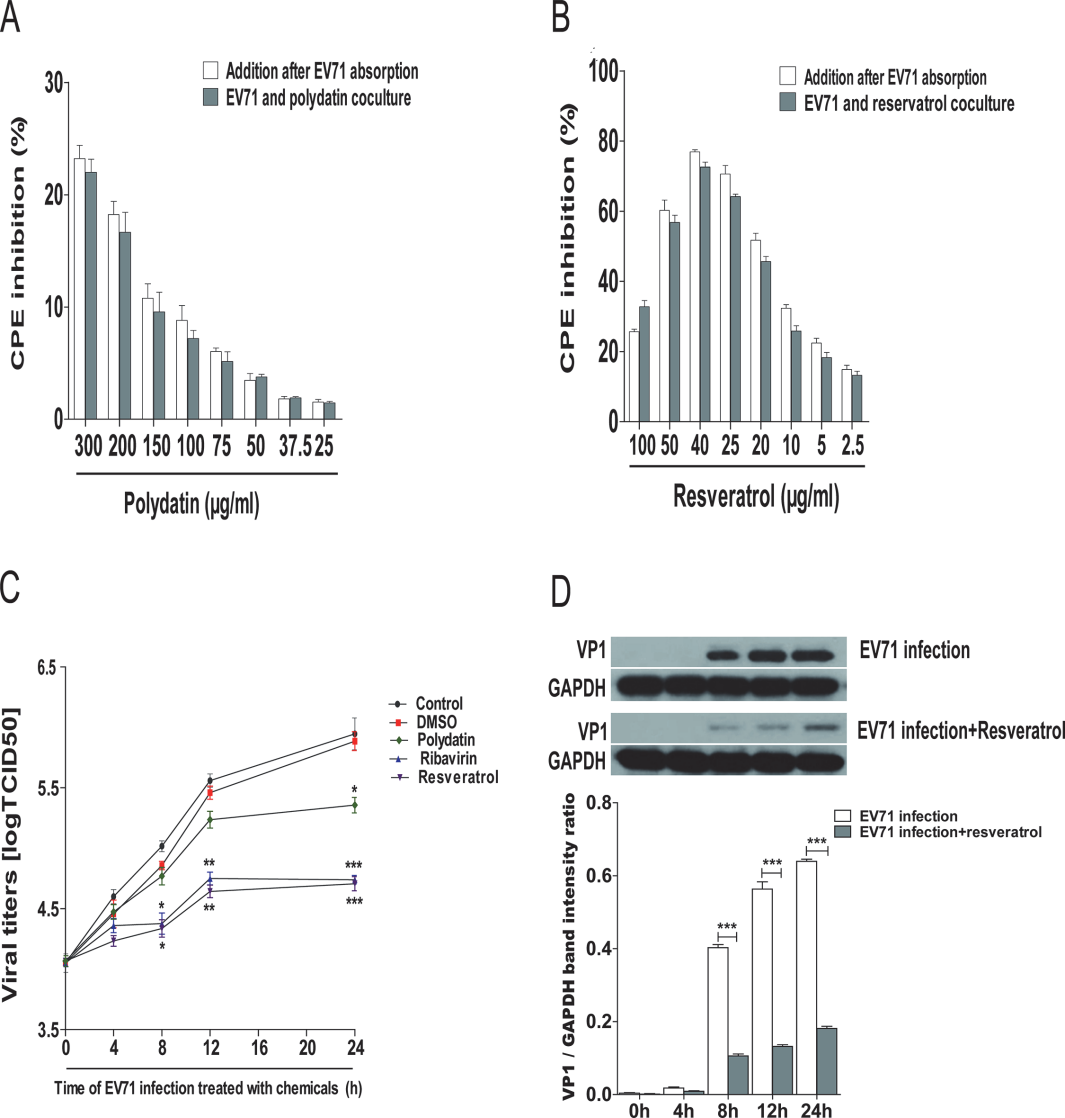
The antiviral effects of polydatin and resveratrol on EV71 replication in RD cells. Polydatin and resveratrol with various concentrations were added at the same time as EV71 or after EV71 absorption for 1.5 h. After incubation for 48 h, MTT assay was performed, and the results were expressed as the inhibitory rate during the cell culture in the presence of drug treatments when compared with the untreated group **(A and B)**. Polydatin (200 μg/ml), resveratrol (30 μg/ml) and ribavirin (125 μg/ml) were added after EV71 absorption for 1.5 h. The culture supernatants were collected at 0, 4, 8, 12, and 24 h p.i., and viral titers (TCID_50_) were calculated using Reed and Muench assay **(C)**. At 0, 4, 8, 12 and 24 h p.i., RD cell lysates were subjected to 10% SDS-PAGE and transferred to PVDF membrane to determine the expression level of EV71/VP1 **(D)**. The data were expressed as mean ± SE from three independent experiments and analyzed by two-way ANOVA with Bonferroni posttests (*p < 0.05, **p < 0.01, ***p < 0.001).

In order to further clarify the correlation between drug treatment modes and EV71 infection, polydatin or resveratrol at various concentrations was added to RD cells at the same time as EV71 or after EV71 absorption for 1.5 h. After incubation for 48 h, the inhibitory rate of polydatin on EV71 replication at the concentration of 300 μg/ml was 23.57 ± 3.64% with IC_50_ of 979.66 μg/ml. No difference in CPE inhibition rate between the virus attachment and co-culture groups was observed (**Fig. 2A)**. Resveratrol could significantly inhibit CPE on EV71 infection. The inhibitory activity of resveratrol on EV71 replication within 48 h revealed a concentration-dependent manner (≤40 μg/ml), and the IC_50_ of resveratrol on EV71 replication was 21.36 μg/ml. Furthermore, resveratrol didn’t show any difference between the virus attachment and co-culture groups either (**Fig. 2B**). Time course experiments were conducted to determine the time point of polydatin and resveratrol for inhibiting EV71 replication. Polydatin (200 μg/ml), resveratrol (30 μg/ml) or ribavirin (125 μg/ml) was added after EV71 absorption for 1.5 h. The results indicated that the addition of ribavirin or resveratrol significantly suppressed EV71 replication at 8, 12 and 24 h p.i. Compared with the control, no difference between resveratrol and ribavirin was observed, while polydatin (200 μg/ml) presented a slight inhibitory effect on EV71 replication at 24 h p.i. (**Fig. 2C**). As mentioned above, the antiviral activity of polydatin was weak, thereby we only further analyzed whether resveratrol could interfere with the protein synthesis of EV71/VP1. Approximately 5 × 10^6^ of RD cell-infected with EV71 (MOI = 2) were treated with resveratrol at the dose of 30 μg/ml. Results indicated that the expression of EV71/VP1 protein remarkably increased at 8, 12 and 24 h p.i., respectively. However, EV71/VP1 was significantly reduced in the presence of resveratrol (30 μg/ml) at 8, 12 and 24 h p.i., suggesting that resveratrol could effectively inhibit viral replication in EV71-infected RD cells (**Fig. 2D**).

### Effect of resveratrol on the phosphorylation of IKKs/NF-κB signaling pathway molecules

**Figure 3:**
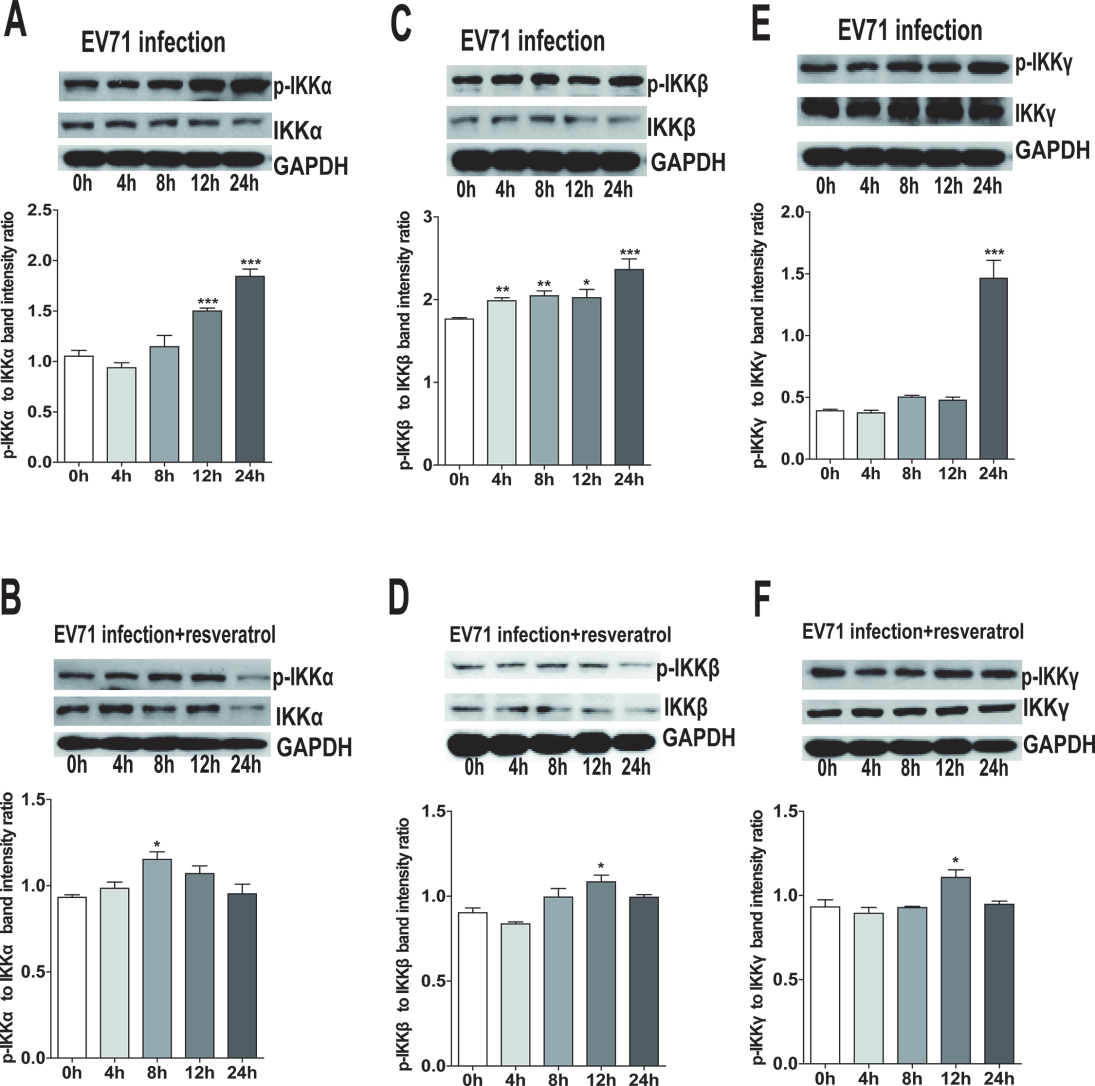
Inhibition of resveratrol on the phosphorylation of IKKα, IKKβ and IKKγ. Western blot analyses of cell lysates from RD cells pre-incubated with or without resveratrol (30 μg/mL) for 1.5 h, and then infected with EV71 (MOI = 2) at 0, 4, 8, 12 and 24 h p.i. were conducted using antibodies against the total or phosphorylated IKKα **(A and B)**, IKKβ **(C and D)**, and IKKγ **(E and F)**, and using GAPDH as the control. The intensity of phosphorylated and total protein bands was quantitated by densitometric analysis and normalized to GAPDH. The data were expressed as mean ± SE from three independent experiments and analyzed by one-way ANOVA followed by a Dunnett's Multiple Comparison Test (*p < 0.05, **p < 0.01, ***p < 0.001).

**Figure 4:**
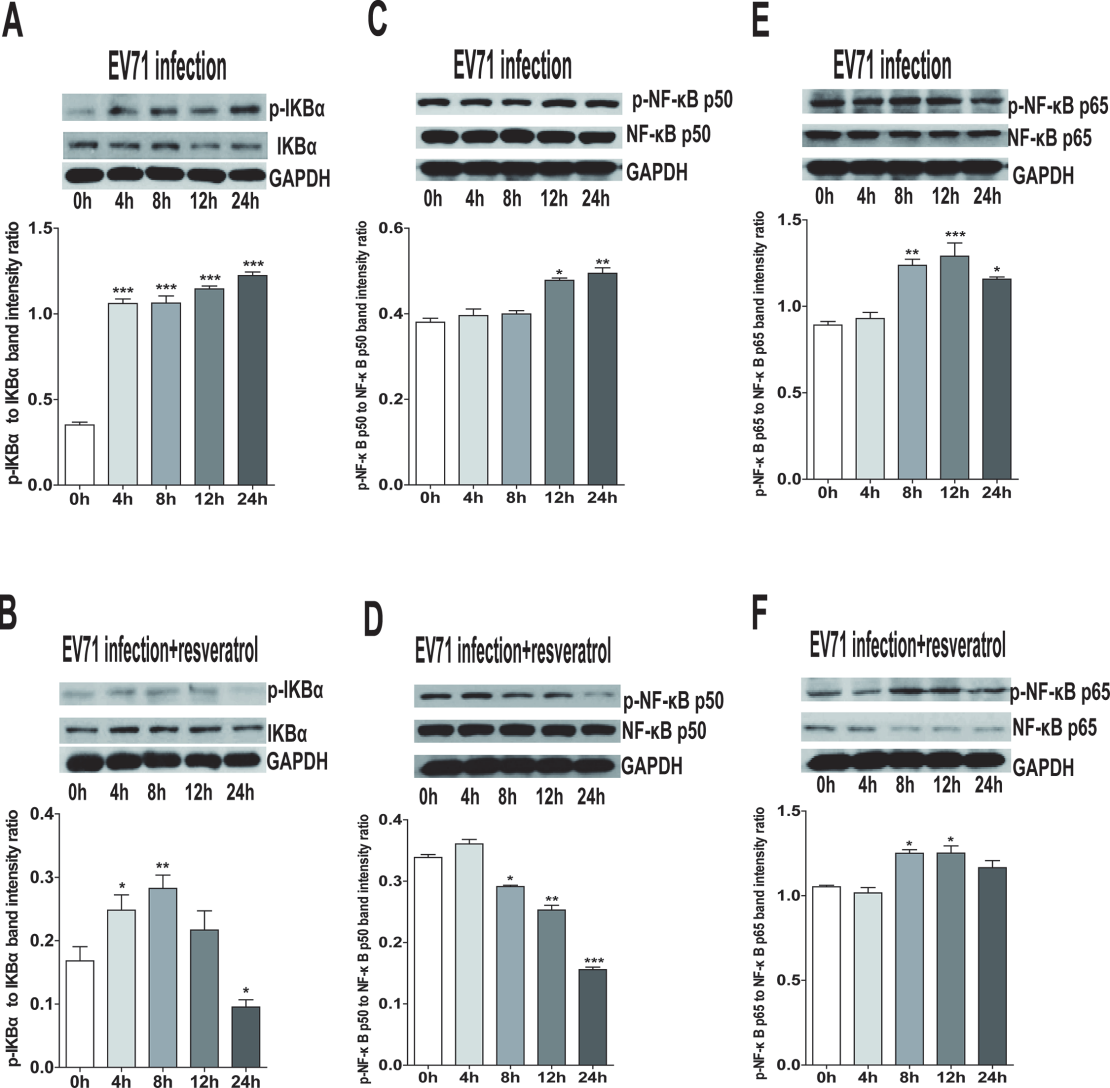
Inhibition of resveratrol on the phosphorylation of IKBα, NF-κB p50, and NF-κB p65. Western blot analyses of cell lysates of RD cells pre-incubated with or without resveratrol (30 μg/ml) for 1.5 h, and then infected with EV71 (MOI = 2) at 0, 4, 8, 12 and 24 h p.i. were conducted using antibodies against total or phosphorylated IKBα **(A and B)**, NF-κB p50 **(C and D)**, and NF-κB p65 **(E and F),** and used GAPDH as the control. The intensity of phosphorylated and total protein band was quantitated by densitometric analysis and normalized to GAPDH. The data were expressed as mean ± SE from three independent experiments and analyzed by One-way ANOVA followed by a Dunnett's Multiple Comparison Test (*p < 0.05, **p < 0.01, ***p < 0.001).

The transcription factor, NF-κB, is considered as one of the key regulators for the inflammatory cellular response, cell proliferation, transformation and tumor progression [17]. During viral infection, although the activation of NF-κB is frequently associated with the protective response of host cells to viral pathogens, some viruses such as EV71, influenza A virus and HSV-1 require the activation of NF-κB signal pathway for efficient viral replication [18–20]. In the present study, EV71 infection promoted the phosphorylated levels of IKKα, IKKβ, and IKKγ in RD cells at 4, 8, 12 and 24 h p.i., but they revealed a significant reduction in the presence of resveratrol (30 μg/ml) **(Fig. 3)**. Meanwhile, the increased phosphorylation levels of IKBα, NF-κB p50, and NF-κB p65 in EV71-infected RD cells were inhibited by resveratrol (30 μg/ml) **(Fig. 4)**. Therefore, resveratrol inhibited EV71-induced activation of IKKs/NF-κB signal pathway, subsequently blocking the nuclear translocation of NF-κB and the secretion of inflammatory cytokines.

### Stimulation of IL-6 and TNF-α secretion during EV71 infection

**Figure 5:**
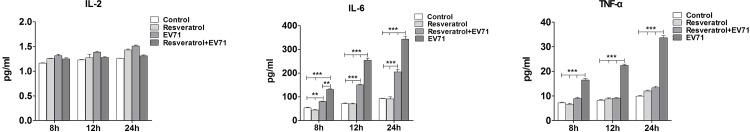
Promotion of IL-6 and TNF-α releases in RD cells with EV71 infection. Control: uninfected RD cells; Resveratrol: uninfected RD cells treated with resveratrol; EV71: EV71 infection; Resveratrol + EV71: RD cells pretreated with resveratrol for 1.5 h before EV71 infection. The culture supernatants were harvested at 8, 12 and 24 h after infection to measure the release of cytokines by luminex fluorescent technique. The data were expressed as mean ± SE from three experiments and analyzed by two-way ANOVA with Bonferroni post-hoctests. *P < 0. 05; **P < 0. 01; ***P < 0. 001. ****

Once infected by viruses, host cells will initiate innate or adaptive immunity, and then secrete a series of proinflammatory cytokines. However, the overexpression of these cytokines usually leads to the damage, chronic inflammation, and other severe immunoresponses, even death of tissues and host cells. In the present study, RD cells were infected with EV71 at a MOI of 2 for 24 h, and no difference between the control and resveratrol groups was observed. Compared to both groups, EV71-infected cells had the increased production of IL-6 and TNF-α at 8, 12 and 24 h p.i. In contrast, the levels of IL-2 did not reveal any change among four groups. The treatment of resveratrol (30 μg/ml) suppressed the secretion of IL-6 and TNF-α in EV71-infected RD cells. Therefore, resveratrol could effectively inhibit the production of inflammatory cytokines and mitigate the damage of RD cells (**Fig. 5**).

## Discussion

Usually, several stages of the viral life cycle are therapeutically targetable as antiviral agents, and virus-encoded proteases and polymerases have been the prominent targets for the development of antiviral drugs [21–23]. However, the effectiveness of antiviral drugs, vaccines and interferons is limited, which correspondingly restricts the complete eradication of viral infection. EV71 is a virus with the characteristics of genetic diversity and rapid evolution. Drug resistance due to viral mutation could be developed soon after chemotherapy. However, traditional Chinese herbs, for example *houttuynia cordata thunb* and chlorogenic acid, have reported treatment efficacy on EV71 infection [24–26]. Polydatin, also named as piceid (3,4’,5-trihydroxystilbene-3-β-D-glucoside), is a monocrystal compound isolated from *Polygonum cuspidatum Sieb*. *et Zucc*. (Polygonaceae) [9, 27]. Resveratrol (3,4,5-trihydroxy-*trans*-stilbene) is a phenolic compound produced by various family members of spermatophytes such as grapes, peanuts and *P*. *cuspidatum*. In recent years, resveratrol has been gained extensive attention due to its wide variety of preventive function for cardiovascular diseases and cancer, and the control function for bacterial and viral infections [28–30]. Moreover, resveratrol usually interferes with signaling pathways in cells to execute its treatment efficacy for a wide variety of virus infections [31].

Previous studies have reported that resveratrol has antiviral effects on HSV, HIV-1, HBV, Epstein-Barr virus, influenza A virus and HCMV [8, 32–36]. However, it is still unknown whether polydatin and resveratrol have the antiviral effect on EV71. Our studies have confirmed no cytotoxicity of polydatin and resveratrol on RD cells at doses of 200 and 31.25 μg/ml, respectively. Meanwhile, the IC_50_ of polydatin and resveratrol are 979.66 and 21.36 μg/ml. Ribavirin is a guanosine (ribonucleic) analog and nucleoside inhibitor used to terminate the synthesis of viral RNA and the capping of viral mRNA [37, 38], indicating that both resveratrol and ribavirin can significantly inhibit the CPE caused by EV71. Although polydatin has been proved to elicit numerous biological effects through its anti-inflammatory and anti-oxidant properties through up-regulating the expression of glioma-associated oncogene homolog1 (Gli1), Patched-1 (Ptch1) and superoxide dismutase 1 (SOD1), as well as down-regulating the expression of NF-κB[39], its antiviral effect on EV71 replication has been validated to be weak in our study. Thus, further studies on antiviral effect and mechanism of polydatin on EV71 are terminated.

The inhibitory activity of resveratrol on EV71 replication is a dose-dependent mode. When resveratrol (30 μg/ml) and ribavirin (125 μg/ml) were added simultaneously with or after EV71 absorption for 1.5 h, the viral titers in cell supernatant revealed a decreasing trend, suggesting that resveratrol may block EV71 replication in RD cells, but independent of viral attachment and penetration. EV71 is a single-stranded positive-sense RNA virus encoding four capsid proteins (VP1, VP2, VP3, and VP4) and seven nonstructural proteins (2A, 2B, 2C, 3A, VPg, 3C, and 3D) [40]. In addition to protecting viral RNA from nuclease cleavage, the capsid proteins such as VP1, VP2, and VP3 are antigenic protein, and can recognize the receptors on the surface of specific host cells [41, 42]. In this study, the protein synthesis of VP1 significantly increased in EV71-infected RD cells at 8, 12 and 24 h p.i., respectively. On the other hand, EV71/VP1 was distinctly reduced in the presence of resveratrol. These results indicated that resveratrol could severely impair EV71 replication and mitigate RD cell damage. Further investigations are required to elucidate the effect and mechanism of resveratrol on EV71 infection.

To date, five proteins belonging to the NF-κB family have been identified in mammalian cells: NF-κB1 (p50), NF-κB2 (p52), RelA (p65), RelB and c-Rel. In the resting condition, the NF-κB p50/p65 heterodimer is in an inactive state in the cytoplasm and binds to the inhibitory molecule IκBα (inhibitor of NF-κB). After the stimuli of bacterial and viral infections, the upstream IKK complex including IKKα, IKKβ and IKKγ can phosphorylate IκBα to result in its release from NF-κB and degradation via ubiquitin proteasome pathway. In recent years, resveratrol has been extensively explored, and demonstrated its role in the regulation of crucial cellular processes such as cell cycle, apoptosis and inflammatory responses [43–45]. In our study, EV71 infection gradually activated the phosphorylation of IKKα, IKKβ and IKKγ, then IKBα, NF-κB p50 and NF-κB p65 were phosphorylated in turn. Subsequently, NF-κB might translocate into the nucleus to bind the promoters of target genes and accelerate the secretion of IL-6 and TNF-α. On the other hand, the level of IL-2 did not reveal the change in EV71-infected RD cells. Subjected to the pretreatment of resveratrol, the phosphorylation of IKKs/NF-kB pathway molecules could be significantly inhibited, while the secretion of IL-6 and TNF-α revealed the reduction in EV71-infected RD cells. According to previous reports, resveratrol is involved in the regulation of many signal pathways such as the pathways of NF-κB, EGFR and SIRT1 activation for antiviral defense in different cells [8, 46, 47]. The activation of NF-κB signal pathway may be efficient for viral replication, such as EV71, influenza A virus and HSV-1. Our results indicate that resveratrol can effectively protect the response of host cells to EV71 infection by targeting NF-κB signal pathway, inhibit inflammatory reaction and extenuate the damage of RD cells.

Presently, EV71 mouse models have been employed to evaluate the anti-EV71 activities of drugs, including pleconaril, ribavirin and rupintrivir[48–50]. Moreover, these anti-viral interventions display certain protective or therapeutic effects when given either before or after EV71 infection that include the reductions in morbidity, mortality, tissue virusloads and the alleviation of lesions. Although resveratrol has little cytotoxicity and shows the inhibitory effect on EV71 replication *in vitro*, the efficiency should be further evaluated on animal models in the follow-up study. In addition, resveratrol can be rapidly metabolized to glucuronide or sulfate compounds with low bioavailability on first pass after oral administration, which seems to be difficult in clinical application. Therefore, resveratrol may serve as a lead for chemical modification and a suitably modified analogue as a clinical candidate.

In summary, EV71 infection can stimulate the phosphorylation of IKKα, IKKβ, IKKγ, IKBα, NF-κB p50 and NF-κB p65, which correspondingly enhances the secretion of IL-6 and TNF-α. Whereas, the activation of the signal pathway and EV71 replication can be blocked by resveratrol, suggesting that it is effective to inhibit EV71 infection through blocking IKKs/NF-κB pathways. The underlying mechanisms is still not clear and needs to be further studied *in vitro* or *in vivo* to highlight IKKs/NF-κB pathway as potential broad antiviral molecular targets for the treatment of EV71 infection.
